# Human Trafficking: The Shameful Face of Migration

**DOI:** 10.1371/journal.pmed.1001047

**Published:** 2011-06-21

**Authors:** The PLoS Medicine Editors

## Abstract

In the June editorial, the *PLoS Medicine* Editors highlight the global importance, impact, and health implications of trafficking in persons. This editorial is part of the *PLoS Medicine* Series on Migration and Health (http://www.ploscollections.org/migrationhealth). The editors argue that despite internationally agreed policies outlining states' responsibilities to protect those affected by trafficking, many countries lack the political will to establish those protections.

This month *PLoS Medicine* publishes a series of articles focused on migration and health. The series provides new insights into the ways by which global movement of people influences the health of individuals and populations, and sets out policy approaches for protecting the health of those most vulnerable during the five phases of migration (http://www.ploscollections.org/migrationhealth). In an introduction to the series, Cathy Zimmerman and colleagues [1] propose a new framework for understanding migration as a series of phases, defining categories of people affected by migration and suggesting estimates of the likely size and importance of each group. One category, that of trafficked persons, stands out as a uniquely vulnerable group that is largely ignored.

Trafficked persons are defined as “individuals who are coerced, tricked or forced into situations in which their bodies or labor are exploited, which may occur across international borders or within their own country” [1]. While the vulnerability of trafficked people is considerable, Zimmerman and colleagues [1] suggest that the true magnitude of the problem is still unknown. Underscoring this point, the latest United Nations report on trafficking highlights a “knowledge crisis,” whereby aggregate statistics cannot be reliably generated, given that trafficking is both highly profitable and one of the world's largest criminal industries [2]. Attempts to understand the scale of the problem are further hampered by differences between countries in defining what constitutes trafficking, in their efforts to protect those exploited by it and prosecute the traffickers, and in reporting data. Despite these difficulties, a recent US Trafficking in Persons Report [3] suggests that the numbers are massive: around 12 million men, women, and children around the world are currently in forced labor, bonded labor, or forced prostitution, with approximately 600,000–800,000 trafficked each year.

Other sources [2],[4] suggest that coercion into the sex trade, overwhelmingly of women and children, comprises the largest proportion of all those trafficked internationally, with a smaller minority trafficked for labor or other forms of exploitation. Such estimates tend to be based around analysis of the very small numbers of cases reported to, or investigated by, national authorities, and it has been suggested that only 0.4% of likely victims of trafficking are ever identified as such [3]. Some authorities attempting to compile a profile of the international picture of trafficking have recognized, however, that aggregate statistics are likely strongly biased towards over-detection of women and girls who have been trafficked into sexual exploitation, and under-detection of individuals trafficked for other reasons, such as for bonded labor, domestic servitude, or as child soldiers [4].

The health implications for those affected by trafficking, and particularly for sexual exploitation, are severe during any phase of migration. Individuals face enormous barriers in many countries in accessing health services and other forms of support, and many health problems or risks arise directly from marginalization, insecurity, and difficulties obtaining care [5]. Guidance for practitioners in providing care for those who have been trafficked highlights the importance of providing “trauma-informed care”—recognizing the myriad of symptoms and presentations that may have been influenced by prior traumatic experiences [5]. This guidance also emphasizes the importance of understanding local referral and protection mechanisms for those who have been trafficked. However, mechanisms differ considerably between countries, and many have no dedicated national referral system for providing coordinated, specialized care for those who are suspected of being trafficked [4]; further, the services available often depend on an individual's cooperation with criminal proceedings to prosecute traffickers in their destination country [6]. Even in high-income countries, authorities have acknowledged that they do not “have victim-sensitive procedures to determine, or to meet the health needs of trafficked women”; these needs are complex and involve cooperation among multiple health, social, and legal services [6]. As a result, clinicians may lack a clear understanding of how best to negotiate these arrangements and protect the health and rights of individuals who they suspect have been trafficked.

Despite the need for a better understanding of the scale and impact of people trafficking worldwide, established international treaties recognize and define states' responsibilities in curbing trafficking and protecting those affected by it. The two Palermo Protocols adopted by the United Nations (see http://www.unodc.org/unodc/en/treaties/CTOC/index.html), and ratified by 117 countries, define states' responsibilities towards the protection of those trafficked and includes the obligation to introduce trafficking legislation. In Europe, the Council of Europe Convention on Action against Trafficking in Human Beings [7] establishes states' duties to prevent trafficking, protect the human rights of victims of trafficking, and to prosecute the traffickers. Shockingly, however, these international treaties are ignored, or not fully supported, by a large number of states. Scores of countries have no, or only partial, criminal legislation covering people trafficking [4]. Even where legislation does exist, prosecutions are rare, and by the time the UN prepared its latest report on global trafficking, over 40% of the world's countries had not recorded a single conviction [4].

The UK has been described as “tier 1” by the US State Department's international rankings [3] in fully complying with the US proposals for minimum standards in protection of trafficking victims. However, despite this apparently high standard, the current government is failing to put its money where its mouth is. The Poppy Project [8], acknowledged by the UNDOC report [4] as providing the UK's major referral and outreach services for trafficked women in the UK, is to have its funding withdrawn. It is not clear whether replacement services will have the expertise needed to provide outreach and help to women in accessing health care, social services, counseling, and reintegration and legal advice. The national referral mechanism in the UK, ostensibly set up to provide protection for those who have been trafficked, has no appeals process if a decision is negative (i.e., it is decided that the individual has not been trafficked). And although the UK government initially indicated it would not sign a new European Union directive on people trafficking, which would allow for traffickers from the EU to be prosecuted in any EU country and afford greater protections for those who have been trafficked, has finally U-turned under public pressure and declared its support [9].

 Eight years ago, the authors of a research study examining the effects of trafficking of women in the EU advocated that trafficking be recognized as a health issue and set out the importance of acknowledging trafficked women's rights to health as a fundamental part of their human rights [6]. There are now established international policy instruments establishing the “three P's” of states' responsibilities: Prevention, Protection (for trafficked peoples), and Prosecution. Despite these policies, the reality is that we still do not know enough about the scale and impact of trafficking, and many countries lack the political will to provide the protection and health-related services that those made vulnerable through trafficking most need.
